# Relationship between lipid accumulation product and oxidative biomarkers by gender in adults from Yucatan, Mexico

**DOI:** 10.1038/s41598-022-18705-8

**Published:** 2022-08-22

**Authors:** Roberto Lugo, Azalia Avila-Nava, Alfredo Geovanny Pech-Aguilar, Isabel Medina-Vera, Martha Guevara-Cruz, Ana Ligia Gutiérrez Solis

**Affiliations:** 1 Hospital Regional de Alta Especialidad de la Península de Yucatán (HRAEPY), Calle 7, No. 433 por 20 y 22, Fraccionamiento Altabrisa, 97130 Mérida, Yucatán Mexico; 2 Departamento de Metodología de la Investigación, Instituto Nacional de Pediatría (INP), 04530 Mexico City, Mexico; 3 Departamento de Fisiología de la Nutrición, Instituto Nacional de Nutrición y Ciencias Médicas Salvador Zubirán (INCMNSZ), 14080 Mexico City, Mexico

**Keywords:** Biomarkers, Medical research, Molecular medicine

## Abstract

Excessive adipose tissue can lead to metabolic abnormalities resulting in lipid alteration and oxidative stress (OS) status. The lipid accumulation product (LAP) index is a biomarker that indicates central lipid accumulation and has been proposed as an accurate and independent indicator of risk for several cardiometabolic related conditions. There is a lack of information about the possible association of LAP and OS biomarkers. Therefore, this work aimed to investigate the relationship between LAP and OS biomarkers in adults. A cross-sectional study was performed in 250 subjects attending the Hospital Regional de Alta Especialidad de la Península de Yucatán. Anthropometrical and clinical parameters were measured. The serum oxidative biomarkers such as malondialdehyde (MDA) and total antioxidant capacity (TAC) were evaluated by spectrophotometry and by the oxygen radical absorbance capacity (ORAC), respectively. A positive and significant correlation between serum levels of MDA and LAP (r = 0.162, p = 0.010) was observed. This relationship was stronger in women (r = 0.189, p = 0.013) than in men. The association between them remained significant after adjusting for confounders (r = 0.23, p < 0.001). A cutoff of LAP of 73.73 predicts high levels of MDA in women aged between 40 and 59. LAP index was associated with OS biomarkers in women and men from Yucatan, Mexico. Therefore, the elevation of the LAP index could identify an imbalance in the redox status.

## Introduction

Excessive adipose tissue can lead to metabolic abnormalities resulting in lipid alteration and oxidative stress (OS) status^1^. These alterations are reported in several cardiometabolic related conditions such as insulin resistance (IR), metabolic syndrome (MetS), type 2 diabetes (T2D), and cardiovascular diseases (CVDs)^2^. The lipid accumulation product (LAP) index is a biomarker that indicates central lipid accumulation and has been proposed as an accurate and independent indicator of risk for the conditions mentioned above. In fact, it has been found in several populations that LAP is a better predictor for MetS and T2D^3^. Because the LAP index is calculated using waist circumference and triglyceride levels^4^, it has a strong correlation with traditional and well-established risk markers^5,6^.

In Mexico, CVDs and T2D are the leading causes of mortality^7^, and MetS is reported to have a high prevalence in several studies among Mexican adults^8^. In particular, all these conditions are found at high frequencies in the Yucatan peninsula population^9^.

An excessive amount of adipose tissue results in a disruption of normal physiological processes, resulting in an imbalance of energy and redox status. Redox imbalance contributes to the generation of OS, and the progression of a pro-oxidative environment, including excessive production of reactive oxygen species (ROS) and a reduction of antioxidants^10^. These alterations promoted the oxidation of biomolecules such as proteins, DNA, and lipids. The lipid oxidation generates hydroperoxides like malondialdehyde (MDA), which is associated with cell and tissue dysfunction^11^. Moreover, MDA is one of the most common measurements for the determination of OS^12^. In fact, studies showed that higher levels of MDA were found in individuals with higher body mass index (BMI) and waist circumference (WC)^13,14^. This suggests that OS promotes the accumulation of oxidized lipids in the adipose tissue that leads to several conditions, including IR, atherosclerosis, and T2D^15–17^. Despite this close relationship between excessive lipid accumulation and OS, there is a lack of information about the possible association of LAP and OS biomarkers. In a previous study by our lab group, LAP was shown to be the most effective indicator for diagnosing MetS in adults^18^. Against this background, the present work aims to investigate the relationship between LAP and OS biomarkers in adults from Yucatan, Mexico.

## Results

A total of 250 adults were evaluated (77 men and 173 women). Men (48.2 ± 11.4) were older than women (46.2 ± 12.5). T2D (67%) and MetS (53%) were found to be more frequent in women than in men. Regarding the clinical characteristics, results showed that men have higher and significant values of WC (p = 0.016), systolic blood pressure (SBP) (p = 0.014), creatinine (p < 0.001), uric acid (UA) (p < 0.001); and low levels of high-density lipoprotein-cholesterol (HDL-C) (p < 0.001) compared to women. However, women reported higher BMI (p = 0.022) and MDA levels (p < 0.001) (Table 1).

**Table 1:** Baseline demographic, comorbidities and clinical characteristics among men (n = 77) and women (n = 173).

Characteristics	Men (n = 77)	Women (n = 173)	*p-*value
Age (y)	50 (42–58)	46.2 (39–55)	**0.001**
**Comorbidities, n (%)**			
T2D	15 (19)	52 (67)	0.082
Hypertension	26 (33)	59 (34)	0.952
MetS	28 (36)	92 (53)	**0.014**
**Clinical**			
WC (cm)	94 (89–101)	91 (83–101)	**0.016**
BMI (kg/m^2^)	29.1 ± 4.53	30.9 ± 6.29	**0.022**
SBP (mmHg)	126 ± 17.3	120 ± 20.5	**0.014**
DBP (mmHg)	73.3 ± 10.8	71.2 ± 10.2	0.150
FPG (mmol/L)	5.22 (4.82–5.77)	5.29 (4.65–6.33)	0.920
Triglycerides (mmol/L)	1.68 (1.28–2.84)	1.31 (1.21–2.28)	0.261
HDL-C (mmol/L)	1.03 (0.86–1.17)	1.15 (1.02–1.37)	** < 0.001**
Urea (mmol/L)	4.56 (3.6–6.58)	4.35 (3.21–5.74)	0.104
Creatinine (µmol/L)	83.1 (76.9–102)	62.77 (55.7–74.3)	** < 0.001**
UA (mmol/L)	359.85 (308–404)	285.5 (238–345)	** < 0.001**
*hs*-CRP (mg/dL)	2.12 (1.32–6.84)	2.49 (1.48–8.13)	0.524
LAP	55.3 (30.8–78.8)	55.5 (34–83.1)	0.803
MDA (nmoles/mL)	17.6 ± 5.77	20.6 ± 6 .84	**0.001**
TAC (micromoles TE/mL)	966 (897–1006)	957 (870–997)	0.187

Data is presented using mean ± standard deviation or median (Interquartile range). Differences between groups were evaluated with independent t-test or Mann–Whitney test.

p-value less than 0.05 was considered statistically significant.

T2D: type 2 diabetes; MetS: metabolic syndrome; WC: waist circumference; SBP: systolic blood pressure; DBP: diastolic blood pressure; FPG: fasting plasma glucose; HDL-C: high density lipoprotein cholesterol; UA: acid uric; *hs*-CRP: high-sensitive C-reactive protein, LAP: lipid product accumulation; MDA: malondialdehyde; TAC: total antioxidant capacity.

Using a Spearman correlation analysis, the relationship between LAP and oxidative biomarkers was assessed. A positive and significant correlation between serum levels of MDA and LAP (r = 0.162, p = 0.010) was reported (Supplemental Fig. S1). This relationship was stronger in women (r = 0.189, p = 0.013) than in men. Serum levels of the antioxidant capacity (TAC) were not shown to have a significant relationship with LAP (Table 2).

**Table 2:** Correlations between LAP and oxidative stress biomarkers malondialdehyde (MDA) and total antioxidant capacity (TAC) among men (n = 77) and women (n = 173).

Oxidative biomarker	Lipid accumulation product					
	Men n = 77		Women n = 173		Total n = 250	
	*r*	*p*-value	*r*	*p*-value	*r*	*p*-value
MDA	0.097	0.401	0.189	**0.013**	0.162	**0.010**
TAC	0.064	0.579	0.079	0.300	0.071	0.264

MDA: malondialdehyde; TAC: total antioxidant capacity.

p-value less than 0.05 was considered statistically significant.

The association between LAP, MDA, and TAC considering some variables that could act as confounders, was performed through a non-parametric multiple linear regression analysis using a rank transformation of LAP. In model 1, the interaction of LAP and MDA among men and women, was analyzed, including the possible effect of age and HDL-C and high sensitivity C-reactive protein (*hs*-CRP) levels. An independent and positive effect of MDA on LAP was observed to be significant in men (p < 0.001) and in women (p < 0.001), this remains significant after adjusting for confounders. In model 2, the interaction of LAP and serum levels of TAC were adjusted and evaluated. Results showed that the association between LAP and TAC (p < 0.001) is not affected by age or HDL-C and *hs*-CRP levels (Table 3).

**Table 3:** Association between LAP and oxidative stress biomarkers (MDA and TAC) adjusted by confounders among men (n = 77) and women (n = 173).

Lipid accumulation product						
Oxidative biomarker	Men n = 77		Women n = 173		Total n = 250	
	β (95%CI)	*p-*value	β (95%CI)	*p*-value	β (95% CI)	*p*-value
**Model 1**	R^2^ = 0.41	** < 0.001**	R^2^ = 0.19	** < 0.001**	R^2^ = 0.23	** < 0.001**
MDA	0.10 (− 0.08,0.29)	0.268	0.20 (0.06,0.33)	**0.006**	0.19 (0.08,0.30)	** < 0.001**
Age	0.10 (− 0.09,0.29)	0.283	0.20 (0.07,0.34)	**0.004**	0.18 (0.07,0.29)	**0.002**
HDL-C	− 0.53 (− 0.71,− 0.34)	** < 0.001**	− 0.35 (− 0.49,− 0.22)	** < 0.001**	− 0.39 (− 0.51,− 0.28)	** < 0.001**
hs-CRP	− 0.46 (− 0.65,− 0.27)	** < 0.001**	− 0.09 (− 0.23,0.04)	0.181	− 0.22 (− 0.33,− 0.11)	** < 0.001**
**Model 2**	R^2^ = 0.43	** < 0.001**	R^2^ = 0.15	** < 0.001**	R^2^ = 0.20	** < 0.001**
TAC	0.17 (− 0.01,0.36)	0.060	0.07 (− 0.07,0.21)	0.357	0.06 (− 0.05,0.17)	**0.002**
Age	0.07 (− 0.11,0.26)	0.440	0.22 (0.08,0.36)	**0.002**	0.18 (0.07,0.29)	0.312
HDL-C	− 0.56 (− 0.74,− 0.37)	** < 0.001**	− 0.34 (− 0.48,− 0.20)	** < 0.001**	− 0.38 (− 0.50,− 0.27)	**0.002**
hs-CRP	− 0.48 (− 0.67,− 0.29)	** < 0.001**	− 0.10 (− 0.24,0.05)	0.181	− 0.22 (− 0.34,− 0.11)	** < 0.001**

LAP: lipid product accumulation; MDA: malondialdehyde; HDL-C: high density lipoprotein-cholesterol*; hs*-CRP: *high-sensitive* C-reactive protein; TAC: total antioxidant capacity.

p-value less than 0.05 was considered statistically significant.

To assess the clinical impact of LAP index on the oxidative biomarkers (MDA and TAC) a binominal regression was performed. It was reported that women aged 40–59 years old have an increased serum level of MDA (above the 50th percentile). Furthermore, a LAP cutoff value of 73.73 with a sensitivity of 54% and specificity of 67% with a Youden Index of 0.29 was established for this women’s group. Meaning that women with a LAP cutoff of 73.73 are more likely to be in an oxidative state (OR 1.01, p = 0.013). For men, no statistical results were found (Table 4).

**Table 4:** Correlation between cut-off points of LAP and oxidative stress biomarkers (MDA and TAC) in men (n = 77) and women (n = 173) by age groups.

Age groups	Oxidative biomarkers	LAP cut-off	Sensitivity (%)	Specificity (%)	Youden Index	OR	*p-*value
**Men**							
20–39	MDA	25.16	0.00	1.00	0.367	1.02	0.386
(n = 17)	TAC	14.74	0.571	0.778	0.286	0.96	0.315
40–59	MDA	30.60	0.708	0.333	0.202	1.05	0.433
(n = 45)	TAC	44.09	0.833	0.333	0.214	1.00	0.891
≥ 60	MDA	47.19	0.429	0.750	0.339	1.10	0.643
(n = 15)	TAC	35.11	0.286	0.750	0.018	0.99	0.355
**Women**							
20–39	MDA	8.61	0.920	0.200	0.080	0.93	0.326
(n = 46)	TAC	14.62	0.273	0.783	0.138	1.00	0.276
40–59	MDA	73.73	0.543	0.673	0.299	1.01	**0.013**
(n = 101)	TAC	17.98	0.130	0.891	0.011	1.00	0.377
≥ 60	MDA	75.43	0.00	1.00	0.363	1.01	0.370
(n = 26)	TAC	67.72	0.800	0.182	0.170	0.99	0.775

LAP: lipid product accumulation; MDA: malondialdehyde; TAC: total antioxidant capacity.

p-value less than 0.05 was considered statistically significant.

## Discussion

In our study, it was found that there was a significant association between LAP and serum levels of MDA; this association was reported to be stronger in women than in men from our study population. Moreover, models exploring the effect of LAP on OS biomarkers showed that the relationship between LAP and MDA and TAC remains independent and significant, even after age, HDL-C and *hs*-CRP adjustment. Binary logistic regression analysis to estimate LAP predictive ability towards high MDA and low TAC values reported that a cutoff of LAP of 73.73 predicts high MDA levels in women aged between 40 and 59.

OS has been involved in the development of several metabolic diseases, including T2D, MetS, and hypertension, among others^19,20^. At the same time, LAP has been shown to have potential in the prediction of these conditions^21^. However, there is little literature on the association of LAP and OS biomarkers. In fact, only one cohort study that investigated the relationship between OS markers and different cardiometabolic risk factors, showing that LAP is independently associated with the prediction of OS parameters such as total protein sulphydryl groups (tSHG) and the ratio of prooxidant-antioxidant balance and tSHG^22^. Contrary to these results, in our study there was found a significant relationship between the LAP index and high levels of MDA, which is an oxidative biomarker. One reason for this could be explained by the high proportion of participants included in the study showed an excess of body weight (84%). This is in line with previous reports, that pointed out an increase in the activity of another OS marker, the enzyme xanthine oxidase (XO) in the population with obesity^23^. Evidence suggests that excessive accumulation of abdominal fat causes dysregulation of metabolic and signaling pathways, including lipid peroxidation^24,25^. Results from our study showed no difference in the antioxidant activity, but an increase in the concentration of MDA was observed. This means that although there is circulating antioxidative activity, it is insufficient to prevent, reduce or eliminate the oxidative damage caused to the lipids. These results are according to a previous report that showed an increase in activity of the antioxidant enzyme superoxide dismutase (SOD), and high MDA levels in subjects with obesity^26^. The relationship between LAP and OS biomarkers (MDA and TAC) was significant in men and women after adjusting for some cofounders such as age, HDL-C and *hs*-CRP.

The LAP index was defined for the first time by Kahn as a lipid over accumulation^4^, and it is an easy and inexpensive marker that can be used in clinical evaluation, especially for low and middle-income countries with limited resources and a high prevalence of non-communicable diseases like Mexico^27^.

The impact of the redox status on the LAP index is an under-researched issue. Therefore, this study presents the first evidence of the relationship between LAP and OS biomarkers among adults by gender in Mexico.

A limitation of the present work is the study design as cross-sectional study; it is not possible to identify whether lipid over accumulation or imbalance in the redox status occurred first. The presented results should be interpreted in terms of association rather than causality.

In conclusion, LAP index was associated with OS biomarkers in women and men from Yucatan, Mexico. Therefore, the elevation of the LAP index could identify an imbalance in the redox status. The implementation of long-term studies will help to comprehend this association and its mechanisms.

## Methods

### Study population

A cross-sectional study was performed in subjects attending the Hospital Regional de Alta Especialidad de la Península de Yucatán (HRAEPY in the Spanish acronym) in Merida, Yucatan. More detail on the sampling is described and published elsewhere^18^. A total of 250 participants conformed by 77 men and 173 women aged between 20 and 65 years were included. Pregnant women and individuals attending the departments of cardiology, neurology, endocrinology, and oncology were excluded. This research project was approved by the Ethics Committee of the HRAEPY (no. CONBIOETICA-31-CEI-002-20170731) with the identification code: 2017-025. In addition, the Helsinki Declaration for medical research involving human subjects guidelines was followed.

### Anthropometrical measurements and clinical parameters

Anthropometric measurements such as body weight, height, and WC were obtained while wearing light clothing and, according to the Lohman method^28^. BMI was calculated by dividing weight by height squared (kg/m^2^). SBP and diastolic blood pressure (DBP) were estimated with an automatic electronic sphygmomanometer (Omron, Japan). Blood pressure was measured after 15 min of rest in a sitting position, with an uncovered right arm and no crossed legs.

Blood samples were collected after 12 h of fasting. Fasting plasma glucose (FPG) (mg/dL), triglycerides (mg/dL), HDL-C (mg/dL), urea (mg/dL), creatinine (mg/dL), UA, (mg/dL) and *hs*-CRP (mg/dL) levels were measured following standard protocols and with a pre-validated kit (COBAS Integra 400 Plus autoanalyzer, Roche Diagnostics). The LAP was calculated following a formula reported by Kahn^4^:

$${\text{LAP in men }} = \, \left[ {\left( {{\text{WC }}\left( {{\text{cm}}} \right){-}{ 65}} \right)({\text{TG}}\left( {{\text{mmol}}/{\text{L}}} \right)} \right]$$

$${\text{LAP in women }} = \left[ {\left( {{\text{WC }}\left( {{\text{cm}}} \right) \, {-}{ 58}} \right) \, ({\text{TG}}\left( {{\text{mmol}}/{\text{L}}} \right)} \right]$$

### Measurement of redox status by oxidative and antioxidant biomarkers

OS was determined using the product of lipoperoxidation (MDA) and the antioxidant capacity (TAC). Serum levels of MDA were measured by a spectrophotometric method at 586 nm wavelength (BioTek Instruments, Winooski, VT)^29^ and expressed by nmoles/mL of sample. Serum levels of TAC were determined by the oxygen radical absorbance capacity (ORAC) assay^30^. The TAC data was calculated and expressed in Trolox equivalents (TE) per mL of sample (BioTek Instruments, Winooski, VT).

### Statistics

The statistical package Jamovi (version 2.25, Sydney, Australia) was used to analyze the data. Clinical and biochemical characteristics were presented as means ± standard deviation (SD) or medians and interquartile range (IQR) for parameters with non-normal distributions. Proportions and corresponding percentages (%) were reported for comorbidities. Normality was evaluated using the Shapiro–Wilk normality test. Continuous variables between groups were compared using the t-test or the Mann–Whitney U test. A Spearman correlation analysis was performed to access the relationship between LAP, MDA, and TAC. A non-parametric multiple linear regression analysis using rank transformation of LAP was performed and evaluated in Model 1 (MDA) and Model 2 (TAC) to adjust some parameters that could act as confounders (age, HDL-C and *hs*-CRP). Using binomial logistic regression analysis, the discriminatory abilities of LAP to predict OS in the population were examined. Cut-off values were based on the Youden Index (sensitivity + specificity − 1). The sensitivity and specificity of cut-off values of LAP were calculated to evaluate OS based on the increased levels of MDA above the 50th percentile and levels of TAC below the 50th percentile. Cut-off values of LAP were grouped by age among men and women (20–39, 40–59, and ≥ 60 years). For all analyses, statistical significance was set at p < 0.05.

### Ethics declaration

The study was conducted in accordance with the Declaration of Helsinki and approved by the Ethics and Research Committees of the Hospital Regional de Alta Especialidad de la Peninsula de Yucatan (protocol code 2017-025 and date of approval 16 May 2018).

### Consent to participate

Informed consent was obtained from all subjects involved in the study.

## Supplementary Information

Supplementary Figure 1.

## Data Availability

Data underlying this work are available upon reasonable request. Requests for data should be addressed to corresponding author.
