# Supplementary material for: Relationship between lipid accumulation product and oxidative biomarkers by gender in adults from Yucatan, Mexico
Source: Sci Rep. 2022 Aug 22;12:14338. doi: 10.1038/s41598-022-18705-8 (PMC9395434; doi:10.1038/s41598-022-18705-8)

**Supplemental Figure 1.** Correlation between lipid accumulation product (LAP) with A. Malondialdehyde (MDA) and B. Total antioxidant capacity (TAC) among adults (n = 250).


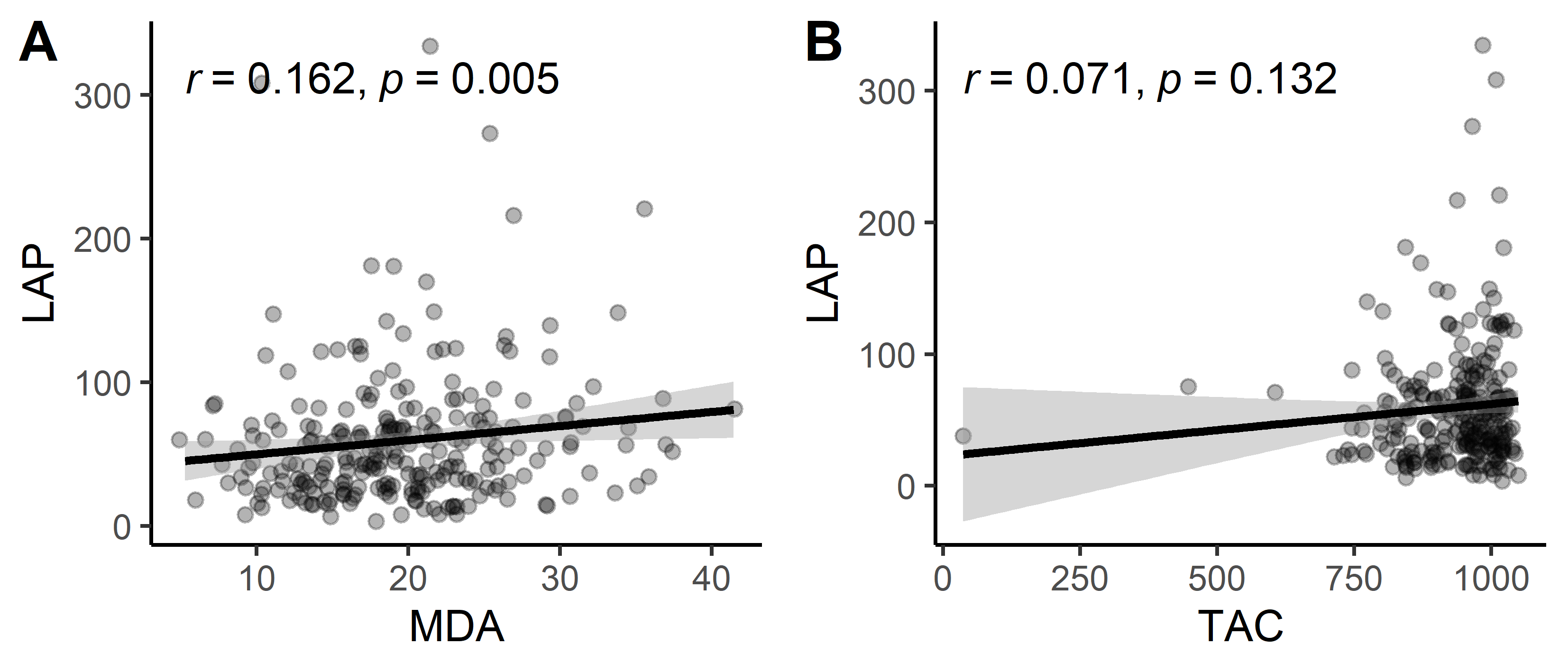

Supplement: Supplementary file 1 — Supplementary Figure 1. [file 41598_2022_18705_MOESM1_ESM.docx]
